# Supplementary material for: The Evolution and Characterization of the RNA Interference Pathways in Lophotrochozoa
Source: Genome Biol Evol. 2024 May 7;16(5):evae098. doi: 10.1093/gbe/evae098 (PMC11114477; doi:10.1093/gbe/evae098)
Supplement: evae098_Supplementary_Data [file evae098_supplementary_data.zip › Supplementary_materials.zip!/selected_miRNAs/NoGe_selected_miRNAs/mirdeep_pdf_files/NMRB01005527.1_Notospermus_geniculatus_strain_Ushimado_scaffold5527,_whole_genome_shotgun_sequence_47688.pdf]

Provisional ID : NMRB01005527.1_Notospermus_geniculatus_strain_Ushimado_scaffold5527,_whole_genome_shotgun_sequence_47688
Score total : 34.8
Score for star read(s) : 3.9
Score for read counts : 29.2
Score for mfe : 2.3
Score for randfold : 0
Score for cons. seed : -0.6
Total read count : 69
Mature read count : 52
Loop read count : 0
Star read count : 17

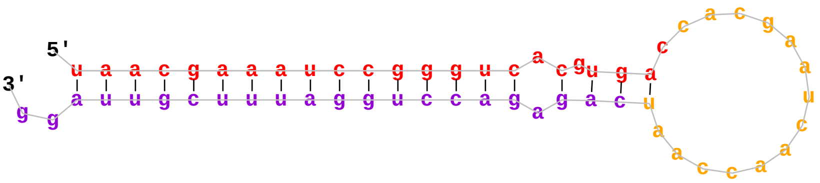

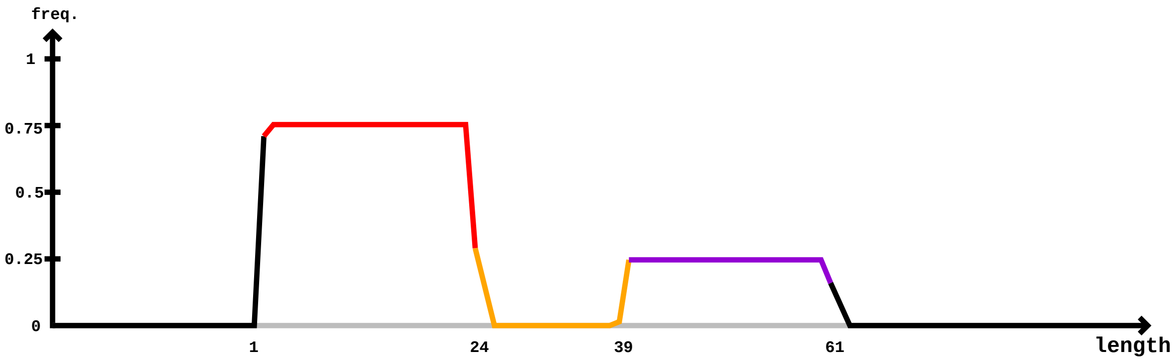

Mature Star

```
5'- cgacguaccuguaccugaccuaacgaaauccgggucacgugaccacgaaucaaccaaucagagaccuggauuucguuaggacagguacaggucagcaucuucggcaaucuuc   -3'   obs
    cgacguaccuguaccugaccuaacgaaauccgggucacgugaccacgaaucaaccaaucagagaccuggauuucguuaggacagguacaggucagcaucuucggcaaucuuc         exp
    .((.(((((((((((((.(((((((((((((((((.(.(((.................)))).))))))))))))))))).)))))))))))..)).)).............   reads   mm   sample
    ....................uaacgaaauccgggucacgugG......................................................................   1       1    Mm1
    ....................uaacgaaauccgggucacguga......................................................................   7       0    Mm1

    ....................uaacgaaauccgggucacguga......................................................................   12      0    Mm2
    ....................uaacgaaauccgggucGcguga......................................................................   1       1    Mm2
    ....................uaacgaaauccgggucacgugG......................................................................   8       1    Mm2
    ....................uaacgaaauccgggucacgugac.....................................................................   5       0    Mm2
    .....................aacgaaauccgggucacgugG......................................................................   1       1    Mm2
    .....................aacgaaauccgggucacgugac.....................................................................   1       0    Mm2
    .........................................................ucagagaccuggauuucguuag.................................   1       0    Mm2
    ..........................................................cagagaccuggauuucguuag.................................   2       0    Mm2
    ..........................................................cagagaccuggauuucguuaU.................................   2       1    Mm2
    ..........................................................cagagaccuggauuucguuagU................................   1       1    Mm2
    ..........................................................cagagaccuggauuucguuagA................................   2       1    Mm2
    ..........................................................cagagaccuggauuucguuagg................................   5       0    Mm2
    ..........................................................cagagaccuggauuucguuagga...............................   1       0    Mm2
    ..........................................................cagagaccuggauuucguuaggU...............................   1       1    Mm2

    ....................uaaGgaaauccgggucacguga......................................................................   2       1    Fm3
    ....................uaacgaaauccgggucacgugac.....................................................................   12      0    Fm3
    ....................uaacgaaauccgggucaGgugac.....................................................................   1       1    Fm3
    .....................aacgaaauccgggucacgugac.....................................................................   1       0    Fm3
    ..........................................................cagagaccuggauuucguuag.................................   1       0    Fm3
    ..........................................................cagagaccuggauuucguuaggU...............................   1       1    Fm3
```
